# Knowledge, Attitudes and Practices of Communal Goat Farmers on the Prevalence and Control of Gastrointestinal Nematodes in Northern KwaZulu-Natal, South Africa

**DOI:** 10.1155/japr/1443083

**Published:** 2025-07-29

**Authors:** Khanyisani Cyril Ndwandwe, Michael Chimonyo, Ana Mbokeleng Tsotetsi-Khambule, Munyaradzi Christopher Marufu

**Affiliations:** ^1^Department of Veterinary Tropical Diseases, Faculty of Veterinary Science, University of Pretoria, Onderstepoort, South Africa; ^2^Department of Animal Science, Faculty of Science, Engineering and Agriculture, University of Venda, Thohoyandou, South Africa; ^3^Department of Life and Consumer Sciences, College of Agriculture and Environmental Sciences, University of South Africa, Roodepoort, South Africa

**Keywords:** diarrhoea, socioeconomic status, the elderly, underdosing, veterinary assistance

## Abstract

Gastrointestinal nematodes (GINs) are a significant impediment to communal goat production, causing considerable economic losses, making their control imperative. The objective of this study was to determine farmers' knowledge, attitudes and practices on the control of GIN in communal goat flocks. A structured close-ended questionnaire was used to conduct face-to-face interviews with communal goat farmers (*n* = 384) across four local municipalities in uMkhanyakude District Municipality. Elderly farmers were 1.4 times more likely to underdose compared to younger farmers (*p* < 0.05). Diarrhoea and emaciation were the most reported clinical signs observed in goat flocks. Farmers were aware of clinical signs of GIN infections (*p* < 0.01) on their farms. However, lack of professional veterinary assistance (*p* < 0.01), low socioeconomic status and low levels of education (*p* < 0.01) reduce the effectiveness of GIN control. Farmer's inability to read manufacturer's instructions was associated with incorrect dosing (*p* < 0.05), which contributed to ineffective management and worsened the impacts of GIN infections. An immediate intervention is required from various stakeholders to achieve sustainable nematode control, with a particular emphasis on women, the elderly farmers and those with low levels of education.

## 1. Introduction

The Southern African goat population has increased from 24.8 million in the year 2000 to 33.4 million in 2017 [1]. Goats contribute to economic, religious and sociocultural enrichment, particularly in resource-limited communal farms. Goats are relatively affordable, can thrive under harsh environmental conditions and have an excellent ability to utilise locally available fibrous feed resources [1–3]. Gastrointestinal nematode (GIN) infections are among the major threats to goat production in tropical and subtropical regions, where warm and humid environmental factors favour their development and transmission [4, 5]. Severe GIN infections in goats can cause diarrhoea, body weight loss, anaemia, bottle jaw, lack of appetite and rough coat [6, 7].

Most farmers depend mainly on anthelmintic drugs to control GIN infections in goats. It is, however, unclear what informs farmer's decision on the selection of anthelmintic agent, drug rotation and identification of goats to treat, timing and frequency of treatment [8]. Resource-poor communal farmers often make these decisions without professional veterinary support. A significant knowledge gap exists regarding the impact of the socioeconomic status of communal goat farmers on the efficiency of GIN control [9]. There is need to ascertain whether these drugs are correctly administered. Resource-poor farmers are likely to be inconsistent in administering anthelmintic drugs [10, 11]. The knowledge, attitudes and practices of farmers are poorly understood in the communal production systems, yet they play a crucial role in the effective control of GIN infections. It is important to know motives behind communal farmer's choice in deworming frequency and choosing the anthelmintic drugs. It also remains unclear whether these drugs are used according to the manufacturer's instructions. Such information could be vital in the development of capacity building programs to improve farmer awareness on sustainable and effective use of anthelmintic drugs. Understanding communal farmers' attitudes on GIN is essential to the development of appropriate prevention and control protocols to combat GIN infections [12, 13]. The execution of these programs requires a clear overview of the attitudes and practices on these farms and an understanding of the factors influencing these attitudes. It was hypothesised that age, education level and income significantly influence farmers' anthelmintic dosing practices to control GIN infections. The objective of this study was to determine knowledge, attitudes and practices on the control of GIN in communal goat flocks.

## 2. Materials and Methods

### 2.1. Study Site

**Figure 1:**
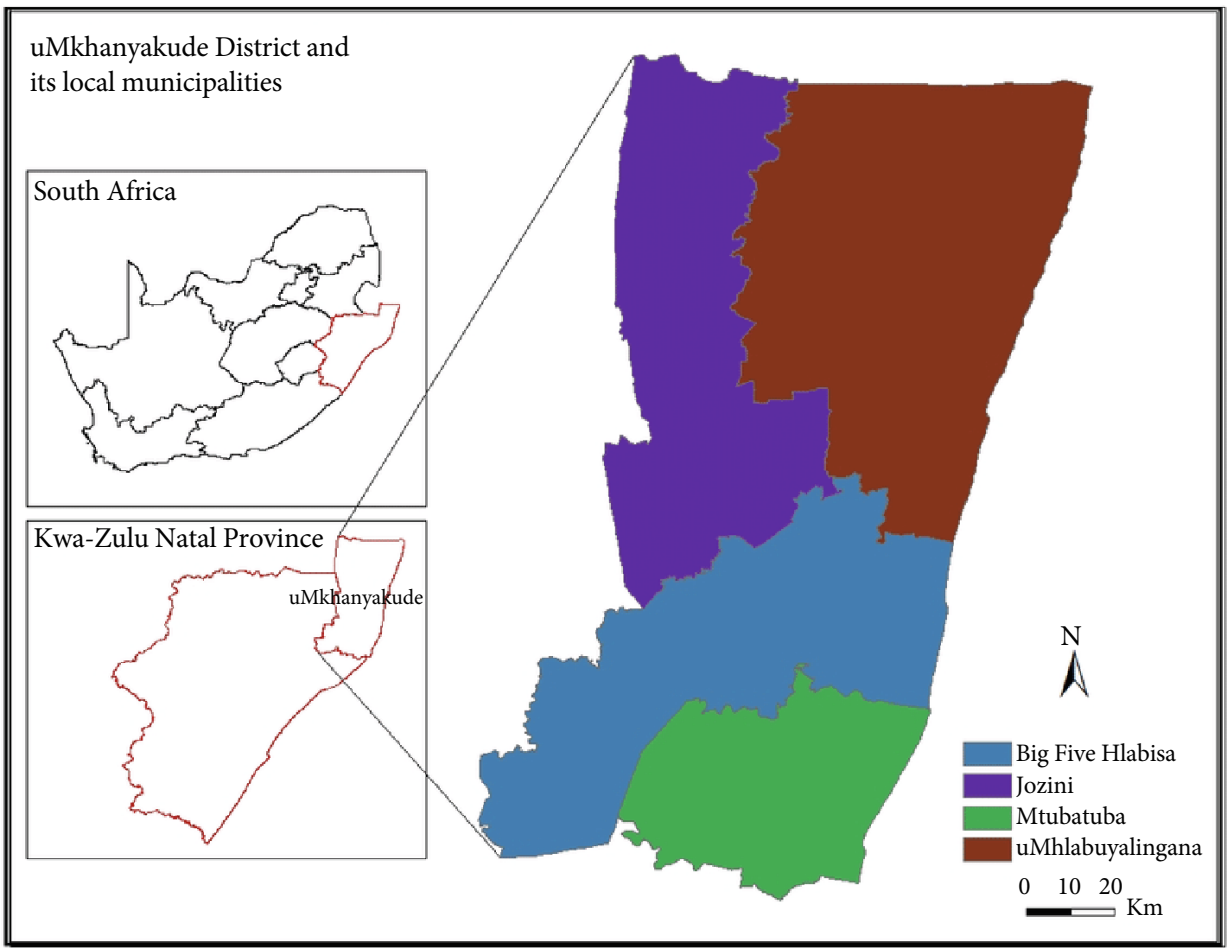
Map showing nine provinces of South Africa with KwaZulu-Natal Province captured in red colour. Locality map depicting four local municipalities where the study was conducted, that is, sky blue represents Big 5 Hlabisa, purple represents Jozini, green represents Mtubatuba and uMhlabuyalingana is represented by the brown colour [14].

The study was conducted at uMkhanyakude District (Figure 1) located at coordinates 27°40⁣′06.9⁣^″^ S and 32°11⁣′48,6⁣^″^ E in Northern KwaZulu-Natal (KZN) [15]. This is the second largest district in KZN and comprises Jozini, uMhlabuyalingana, Hluhluwe Big 5 and Mtubatuba local municipalities. It has a population size of 625,846 and covers an area of 12,818 km^2^ with 151,245 households [16]. uMkhanyakude District is home to 5164 livestock famers with a population of 21,786 cattle and 40,916 goats [17].

uMkhanyakude District experiences four seasons, namely, hot-wet, post-rainy, cool-dry and hot-dry season. The district experiences an average high temperature of 28°C, which lasts for 3 months from December to February and an average low temperature of 19°C, which lasts from June to mid-August. Summer annual rainfall varies from 671 to 1 002 mm, where January, February and March are recorded as the wettest months, while June, July and August have the least annual rainfall [18].

### 2.2. Farmer Selection

Inclusion of farmers in the survey depended on their willingness to participate, being at least 18 years of age and owning a minimum of six goats in their flock. Farmers were selected using snowball sampling, which started with a few known dip tank leaders and willing farmers with the required minimum number of goats and expanded through their referrals. This sampling method is convenient, but it may have excluded farmers without active social connections and introduced bias. However, this was partially mitigated by using multiple starting points (seeds) within each municipality, ensuring diversity in the initial sample and continuous monitoring of the demographic composition of our sample throughout data collection to ensure adequate representativeness. Consent was sought from all participants. A total of 384 goat farmers from four municipalities were interviewed to assess their knowledge, attitudes and practices on the control of GIN in communal goat flocks in uMkhanyakude District.

### 2.3. Data Collection

A pretested structured questionnaire designed in English and translated to the local IsiZulu language, for ease of understanding by respondents, was used to collect data. Trained enumerators administered the questionnaire face-to-face. Pretesting was performed on a group of 10 farmers, three animal health technicians and two agricultural extension officers to ascertain technical soundness, linguistic accuracy and validity before commencement of the study. The questionnaire demonstrated acceptable internal consistency (Cronbach's *α* = 0.74) following a pilot test with 10 farmers from the study area. The pilot testing also resulted in refinement of question wording and terminology to ensure appropriateness for the communal farming context in uMkhanyakude District, enhancing both the clarity and cultural relevance of the instrument. The questionnaire captured farmers' socioeconomic and demographic data, clinical signs of GIN infection, dosing practices and GIN control.

### 2.4. Statistical Analyses

Data were analysed using the Statistical Package for Social Scientists (SPSS Version 26). Five incomplete questionnaires (< 2% of total) were excluded from final analysis. No extreme outliers requiring removal were identified, and all data points fell within reasonable ranges for the study population. Associations between categorical demographic (gender, education level, marital status and local municipality) and response (knowledge and practices) variables were determined using chi-square tests. An ordinal logistic regression model (PROC LOGISTIC) was used to predict the odds of farmers' attitudes toward GIN control in goats. The variables that fitted in the logit model included gender, marital status, age, level of education and level of income. The model used was:

 $$\begin{array}{c} \text{Ln }\left[\frac{p}{1}-p\right]=\beta_{0}+\beta_{1}X_{1}+\beta_{2}X_{2}+\beta_{3}X_{3}+\cdots +\beta_{t}X_{t}+\varepsilon , \end{array}$$

where *p* is the probability of a farmer's perceived knowledge of GIN infection, [*p*/1 − *p*] is the odds ratio of farmer's likelihood to control GIN, *β*_0_ is the intercept, *β*_1_ ⋯ *β*_*t*_ are the regression coefficients of predictors, *X*_1_ ⋯ *X*_*t*_ are the predictor variables and *ε* is the random residual error. When computing each predictor (*β*_1_ ⋯ *β*_*t*_), the odds ratio for controlling GIN was interpreted as the proportion of a farmer controlling GINs versus those that cannot control GINs. A similar model was performed to determine farmer perceptions on the control of GIN infection, with *p* being the probability of a farmer being able to control GIN infection.

## 3. Results

### 3.1. Association Between Sociodemographic Characteristics and Awareness of Farmers on GINs

The sociodemographic characteristics of the respondents are shown in Table 1. Gender was significantly associated with knowledge of GIN awareness, with males demonstrating higher (*p* < 0.05) knowledge across most categories. Awareness of how goats are infected, clinical signs, infected organs, treatment and prevention were all significantly higher among male than female farmers. There was, however, no significant association between gender and knowledge of prevention of GIN infection.

**Figure 2:**
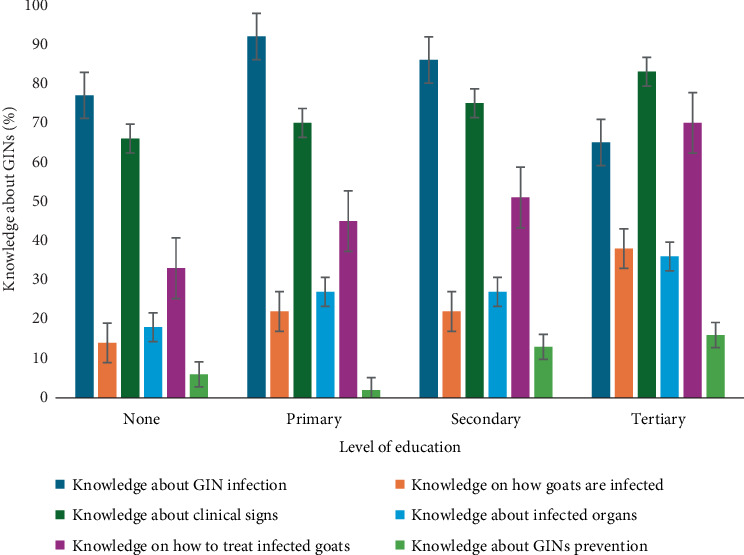
Association between the level of education and communal farmer's knowledge about GIN infections.

Education level showed complex relationships with GIN knowledge. Farmers with higher educational levels had increased awareness of how goats are infected and treated (Figure 2). On the contrary, illiterate farmers and those with primary education had higher awareness of GIN infection signs compared to more educated farmers.

There were significant differences in knowledge of clinical signs, infected organs and prevention methods observed across different municipalities (Table 2). Jozini consistently showed the highest farmer awareness levels, followed by Mtubatuba, while Hluhluwe Big 5 municipality farmers were less informed about affected organs. Prevention knowledge was particularly limited across all municipalities, with only a small percentage of farmers aware of preventive procedures. Age of farmer was not significantly associated with GIN awareness, with all farmer age groups having similar (*p* > 0.05) knowledge of GINs across all knowledge categories.

Marital status emerged as a significant factor in GIN awareness patterns. Married farmers consistently demonstrated higher (*p* < 0.05) knowledge levels compared to single and widowed farmers, particularly in general GIN awareness, infection mechanisms, organ involvement and prevention strategies. There were, however, no significant associations between marital status and knowledge of clinical signs and how to treat the GIN infections.

Clinical sign recognition showed gender-specific patterns, with male farmers significantly better at identifying fever, bottle jaw and lack of appetite than female farmers (Table 3). However, both genders showed equally high recognition rates for more obvious signs like diarrhoea and emaciation.

The logistic regression analysis revealed several concerning practices (Table 4). Older farmers and females showed increased likelihood of using inadequate dewormer dosages, while lower income farmers were more likely to use expired medications. Geographic location also influenced practices, with uMhlabuyalingana farmers showing significantly higher risks for both inadequate dosing and expired drug use. Conversely, less educated and lower income farmers showed greater reliance on medicinal plants.

### 3.2. Farmer's Practices in GIN Infection Control

Professional consultation and information-seeking behaviours showed clear demographic patterns (Table 5). Male farmers and those with tertiary education demonstrated significantly higher rates of veterinary consultation and manufacturer instruction compliance. The education gradient was particularly pronounced, with 97% of tertiary-educated farmers always reading instructions compared to only 35% of those with no formal education.

**Figure 3:**
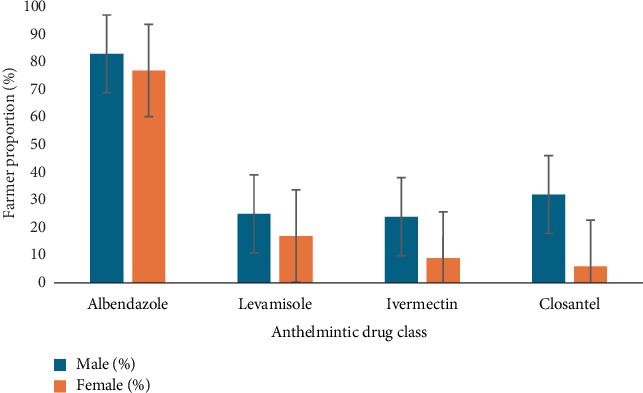
Anthelmintic drug classes used by farmers to control GINs.

Anthelmintic drug classes used by farmers are summarised in Figure 3. There were no significant differences (*p* > 0.05) between gender and using albendazole and levamisole. More male farmers used ivermectin and closantel in comparison to females.

Drug withdrawal compliance revealed significant educational level patterns (Table 6). Tertiary-educated farmers showed the highest withdrawal compliance (80% always observing withdrawal periods) compared to lower rates among uneducated farmers (32%). Seasonal deworming preferences and responses to drug ineffectiveness showed age and educational level–related differences. Younger farmers (18–30 years) predominantly practised autumn deworming and were more likely to increase dosages when drugs appeared ineffective, while older farmers showed more conservative approaches and a greater tendency to seek veterinary advice. More farmers with tertiary education consulted a veterinarian than increased the drug dosage, whereas more farmers without formal education increased the drug dosage than consulted a veterinarian.

## 4. Discussion

Understanding communal farmers' knowledge, attitudes and practices is very crucial in developing sustainable and effective GIP control measures. This study revealed demographic trends with critical implications for GIN control efficacy, particularly that the majority of goat farmers were male, most likely due to the physical demands of livestock farming which align with the traditional male role, consistent with findings reported by Olaogun et al. [19], Jansen et al. [20] and Ndlela et al. [10] from various regions in South Africa. More than 50% of the communal farmers were above 50 years of age with only 18% having attained tertiary education, aligning with Mtshali et al. [21] who highlighted the aging farming population in South Africa, raising concerns about the future of the agricultural industry, as the transfer of indigenous knowledge to younger generations is limited. The reliance of 36% of households on social grants, coupled with limited formal education and financial instabilities, poses significant barriers to improving communal farming practices and productivity [22].

Farmers across four municipalities in the present study were aware of the threat posed by GIN infections on their goat flocks, yet their knowledge was limited to observing late-stage clinical signs such as diarrhoea and emaciation in accord with reports of Emsley [23] in the North West Province of South Africa. Rumosa Gwaze et al. [24] reported similar awareness of communal farmers of the negative impact of internal parasites but also noted their lack of detailed understanding. Research in Gauteng and Limpopo provinces by Tsotetsi et al. [5] and Mphahlele et al. [13], respectively, also reported communal farmers' awareness of GIN infection and failure to recognise other important symptoms such as bottle jaw which Jansen et al. [25] found to be reported commonly by Eastern Cape communal goat farmers.

The deficiencies in farmers' knowledge in the present study were significant because diagnosing GIN infections based solely on clinical signs is insufficient for effective treatment [26]. Lester and Matthews [27] emphasised the importance of modern diagnostic methods, such as the McMaster technique which provide accurate faecal egg counts in early infections. However small scale farmers often cannot afford these methods due to high cost of equipment and labour [28]. Furthermore, the FAMACHA method, a simple tool for assessing anaemia caused by *Haemonchus contortus* infections, was underutilised in the present study, with only a few farmers employing it before administering medication.

Anthelmintic usage was widespread in the present study, with 51% of respondents administering drugs to treat helminth infections, indicating a reliance on chemical control. Nonetheless, many farmers were unaware of best practices such as the use of FAMACHA scoring for targeted selective treatment [29]. This approach could ensure the cost-effective use of anthelmintic drugs while retarding the development of anthelmintic resistance, a global problem faced by livestock farmers [30]. The financial constraints faced by the majority of communal farmers and the inaccessibility of formal veterinary services in the present study limited their access to formal veterinary drugs, forcing them to rely on traditional medicinal plants to control GIN infections [31]. Mavungu et al. [32] in Southern Democratic Republic of Congo reported that 27 plant species were used by communal goat farmers to control GIN infections.

Meanwhile in uMkhanyakude District, more than 65% of farmers used traditional medicinal plants, for example, *Volkameria glabra* (umqoqongo), *Aloe marlothii* (inhlaba), *Cissus quadrangularis* L. (inhlashwana) and *Tetradenia riparia* (iboza) to treat internal parasites [33]. These practices were often driven by the unaffordability of conventional anthelmintics, especially among female farmers who were twice as likely as males to administer less dewormer per goat in an attempt to stretch the limited resources.

The lack of formal education was strongly associated with improper use of anthelmintics, as farmers without formal education were unable to read manufacturer's instructions or contact a veterinarian before administering anthelmintic drugs. Frequent underdosing of anthelmintic drugs (reported by 40% of farmers) could accelerate resistance [30]. This was also noted in Ethiopia where farmers used inappropriate doses and expired drugs in their goat flocks due to lack of veterinary support therefore increasing the need for training programs on precise drug use, particularly correct dosing, among communal farmers [34]. Zvinorova et al. [7] ranked GIP among the top challenges facing communal goat production, attributing this to poor veterinary knowledge among communal farmers. Veterinary consultation in the present study was low especially among female farmers (over 60% did not seek veterinary advice), who often manage goats, a species considered lower value compared to cattle, and deprioritised in terms of veterinary care [24, 35].

Communal farmers in uMkhanyakude District often purchase anthelmintic drugs from the informal market, relying on vendor instructions due to the unavailability of weighing scales for accurate dosing. They rely on the instructions issued by the retailer in the open markets where they buy dewormers. Similar conditions were noted by Siyoum et al. [36] who additionally reported poor storage facilities (exposure to sun) combined with incorrect dosing in open-market drugs. This study also showed that communal farmers in uMkhanyakude District use conventional drugs to control parasite infections. These drugs are purchased in local informal drug retailers, as formal veterinary outlets are not easily accessible, mainly for farmers from Jozini and uMhlabuyalingana local municipalities. Exploitation of conventional drugs was more associated with male than female farmers in all anthelmintic drug classes. Among the commonly used anthelmintic drug classes were albendazole and macrocyclic lactones, with 76% and 35%, respectively, while levamisole and closantel were the least used drugs, with 20% and 3%, respectively. The predominant use of albendazole observed in this study may be attributed to its widespread availability in affordable generic formulations, making it the most economically accessible option for resource-constrained farmers. This cost-driven preference appears to be a broader regional pattern, as demonstrated by Emsley [23] in the North West Province, where 82% of farmers utilised albendazole compared to only 15% using levamisole.

The present study identified a significant disregard of withdrawal periods, with farmers frequently neglecting or being unaware of the need for adherence. Farmers with tertiary education (80%) adhered to anthelmintic drug withdrawal periods, while more than half of the uneducated farmers did not. Most farmers above 60 years of age had no idea of the anthelmintic drug withdrawal periods, while others mentioned that they have witnessed no physical harm after consumption of meat and milk products without adhering to withdrawal periods. Olasoju et al. [37] in Nigeria previously highlighted that knowledge and practices of farmers with regard to drug withdrawals were very poor among illiterate farmers. The consumption of milk and meat products with drug residues may impair human health [38].

Most of the youth (18–30 years) and older (above 61 years) respondents increased the dosage while most middle-aged respondents (41–50 years) consult a veterinarian when the administered drug is not effective. Very few youth and old-age farmers opted to consult a veterinarian if the administered drug was not effective. These results are in contrast with Sazmand et al. [39] in Iran where 95% of the respondents opted to consult a veterinarian and only 38% increased the drug dosage. Such differences suggest varying attitudes toward professional veterinary assistance amongst farmers of different cultures and socioeconomic statuses.

Seasonal patterns of GIN infections were evident, with over 60% of farmers reporting peak infections in summer, while 23% reported deworming their flocks twice a year and 68% deworm whenever they witness infection signs. These findings are supported by Emsley [23] that 63% of farmers at Dr Ruth Segomotsi Mompati District in the North West Province experienced parasite infections in summer. Summer seasons in tropical areas are characterised by warm and wet environmental conditions, which support the hatching and spread of GIN [8, 25, 40, 41]. Apart from conventional anthelmintic drugs, over 65% of farmers alternatively used medicinal plants to control parasite infections, corroborating findings from Jozini Municipality on the extensive reliance on ethnobotanical methods [15]. Over 98% of farmers do not quarantine new stock in their flocks, in stark contrast to quarantine protocols reported by Msimang et al. [42] in the Free State and North West provinces, but supporting reports of lack of quarantine from Thailand [43].

## 5. Conclusions

This study highlights the persistent awareness and attitudinal challenges that communal goat farmers in uMkhanyakude face in effectively managing GIN. Despite general awareness among male farmers, GIN prevention and control are hindered by a combination of old age, low education levels and poor socioeconomic status, leading to ineffective farm management and worsened GIN impacts. Immediate and targeted interventions are required from various stakeholders to achieve sustainable nematode control, with a particular emphasis on the female, illiterate and elderly farmers. More research to streamline the effective communication process between veterinarians and communal farmers, focusing on the sustainable GIN control protocols, is recommended.

## Figures and Tables

**Table 1:** Association between gender and education level with farmers' awareness of the gastrointestinal nematode infections.

**Factor**	**Knowledge about GIN infection (%)**	**Knowledge on how goats are infected (%)**	**Knowledge about clinical signs (%)**	**Knowledge about infected organs (%)**	**Knowledge on how to treat infected goats (%)**	**Knowledge about GIN prevention (%)**
Gender						
Male (*n* = 289)	91	29	81	34	57	13
Female (*n* = 95)	75	7	55	6	27	3
*χ*^2^	17.36	18.09	27.91	28.56	26.43	8.06
*p* value	⁣^∗∗^	⁣^∗∗^	⁣^∗∗^	⁣^∗∗^	⁣^∗∗^	NS
Education level						
None (*n* = 66)	77	14	66	18	33	6
Primary (*n* = 64)	92	22	70	27	45	2
Secondary (*n* = 185)	86	22	75	27	51	13
Tertiary (*n* = 69)	65	38	83	36	70	16
*χ*^2^	10.29	11.68	2.66	5.87	19.49	11.38
*p* value	⁣^∗^	⁣^∗^	NS	NS	⁣^∗∗^	⁣^∗^

Abbreviations: *χ*^2^, chi-square; NS, not significant.

⁣^∗^*p* < 0.05 significant difference. ⁣^∗∗^*p* < 0.01 significant difference.

**Table 2:** Association between municipality and marital status with farmer awareness of gastrointestinal nematode infections.

**Factor**	**Knowledge about GIN infection (%)**	**Knowledge on how goats are infected (%)**	**Knowledge about clinical signs (%)**	**Knowledge about infected organs (%)**	**Knowledge about how to treat infected goats (%)**	**Knowledge about GIN prevention (%)**
Municipality						
Jozini (*n* = 95)	92	22	75	39	49	8
Hluhluwe Big 5 (*n* = 97)	83	18	69	15	43	7
Mtubatuba (*n* = 96)	88	23	79	31	46	19
uMhlabuyalingana (*n* = 96)	83	32	78	24	62	8
*χ*^2^	3.40	5.11	3.28	14.90	8.67	8.92
*p* value	NS	NS	NS	⁣^∗^	⁣^∗^	⁣^∗^
Marital status						
Single (*n* = 158)	84	18	73	27	47	8
Married (*n* = 185)	92	28	79	31	54	13
Widowed (*n* = 36)	75	16	58	8	42	3
Separated (*n* = 5)	80	40	80	40	20	20
*χ*^2^	9.92	9.10	7.17	10.31	4.48	9.36
*p* value	⁣^∗^	⁣^∗^	NS	⁣^∗^	NS	⁣^∗^

Abbreviations: *χ*^2^, chi-square; NS, not significant.

⁣^∗^*p* < 0.05 significant difference.

**Table 3:** Association between gender and clinical signs witnessed by farmers.

**Symptoms**	**Gender**		**χ** ^2^	**p** ** value**
	**Male (%)**	**Female (%)**		
Diarrhoea	99	97	0.24	NS
Fever	44	25	6.34	⁣^∗^
Emaciation	98	97	0.03	NS
Bottle jaw	58	44	6.22	⁣^∗^
Anaemia	27	16	3.70	NS
Lack of appetite	14	5	8.57	⁣^∗^

Abbreviations: *χ*^2^, chi-square; NS, not significant.

⁣^∗^*p* < 0.05 significant difference.

**Table 4:** Odds ratio estimates for practices used by farmers in controlling parasite infections.

	**Predictor**	**Odds ratio**	**L** **CI**	**U** **CI**	**SE**	**p** ** value**
Use less dewormer per goat	Gender	1.975	1.166	3.344	0.269	⁣^∗^
	Age	1.426	1.134	1.793	0.117	⁣^∗^
	Marital status	0.453	0.293	0.703	0.224	⁣^∗∗^
	Educational level	0.952	0.726	1.250	0.139	NS
	Monthly income	1.021	0.796	1.309	0.127	NS
	Jozini	1	—	—	—	⁣^∗∗^
	Hluhluwe Big 5	2.3	1.279	4.107	0.295	⁣^∗^
	Mtubatuba	1.2	0.657	2.044	0.290	NS
	uMhlabuyalingana	2.8	1.558	5.175	0.306	⁣^∗∗^

Use medicinal plants	Gender	0.928	0.262	0.555	1.552	NS
	Age	1.062	0.119	0.842	1.340	NS
	Marital status	0.782	0.222	0.506	1.210	NS
	Educational level	0.755	0.143	0.571	0.999	⁣^∗^
	Monthly income	0.718	0.131	0.556	0.927	⁣^∗^
	Jozini	1	—	—	—	⁣^∗^
	Hluhluwe Big 5	0.508	0.281	0.919	0.302	⁣^∗^
	Mtubatuba	0.726	0.407	1.293	0.295	NS
	uMhlabuyalingana	0.498	0.274	0.905	0.305	⁣^∗^

*Note:* The higher the odds ratio, the stronger the predictions to control GIN infestation. NS: *p* > 0.05. Gender (male or female), age (youth or adult), marital status (single or married), educational level (educated or uneducated) and monthly income (rich or poor).

Abbreviations: LCI, lower confidence interval; UCI, upper confidence interval.

⁣^∗^*p* < 0.05. ⁣^∗∗^*p* < 0.01.

**Table 5:** Association between gender and education level with communal farmers' practices with regard to gastrointestinal nematode control.

	**Consult a veterinarian (%)**					**Read manufacture instructions (%)**				
	**A**	**U**	**S**	**R**	**N**	**A**	**U**	**S**	**R**	**N**
Gender										
Male	28	8	14	5	45	68	18	9	2	3
Female	15	7	8	7	63	51	29	12	1	7
*χ*^2^	13.08					13.67				
*p* value	⁣^∗^					⁣^∗^				
Education level										
None	12	6	15	4	63	35	29	7	8	21
Primary	18	9	13	5	55	46	30	12	2	10
Secondary	26	6	10	7	51	70	21	8	0	1
Tertiary	42	12	16	3	27	97	2	1	0	0
*χ*^2^	29.50					87.04				
*p* value	⁣^∗^					⁣^∗∗^				

Abbreviations: *χ*^2^, chi-square; A, always; N, never; NS, not significant; R, rarely; S, sometimes; U, usually.

⁣^∗^*p* < 0.05 significant difference. ⁣^∗∗^*p* < 0.01 significant difference.

**Table 6:** Farmer's perceptions relating to administration of anthelmintic drugs.

**Practices**		**Farmer's age**					**Education level**			
		**18–30**	**31–40**	**41–50**	**51–60**	**≥61**	**None**	**1** ^**st**^	**2** ^**nd**^	**3** ^**rd**^
Consider meat and milk withdrawals (%)	Yes	24	47	64	49	35	32	28	44	80
	Sometimes	31	25	21	20	19	14	32	24	13
	Never	45	28	15	31	46	54	40	32	7

*χ* ^2^	35.27						65.11			

*p* value	⁣^∗^						⁣^∗∗^			

Administered drug not effective (%)	Increase dose	64	51	36	56	43	58	51	53	28
	Contact vet.	10	18	40	30	21	13	12	23	45
	Change the drug	26	30	24	17	36	29	36	25	28

*χ* ^2^	24.30						31.46			

*p* value	⁣^∗^						⁣^∗∗^			

Deworming season (%)	Winter	11	4	9	5	3	8	2	1	2
	Summer	19	20	23	36	33	20	24	21	42
	Autumn	60	66	48	49	56	68	64	65	42
	Spring	10	11	19	10	8	4	11	14	14

*χ* ^2^	24.72						19.16			

*p* value	⁣^∗^						⁣^∗^			

Abbreviations: *χ*^2^, chi-square; 1^st^, primary school level; 2^nd^, secondary school level; 3^rd^, tertiary school level; NS, not significant.

⁣^∗^*p* < 0.05 significant difference. ⁣^∗∗^*p* < 0.01 significant difference.

## Data Availability

The data that support the findings of this study are available from the corresponding author upon reasonable request.
